# Mapping Tumor Heterogeneity via Local Entropy Assessment: Making Biomarkers Visible

**DOI:** 10.1007/s10278-023-00799-9

**Published:** 2023-02-27

**Authors:** Guido Costa, Lara Cavinato, Francesco Fiz, Martina Sollini, Arturo Chiti, Guido Torzilli, Francesca Ieva, Luca Viganò

**Affiliations:** 1 Division of Hepatobiliary and General Surgery, Department of Surgery, IRCCS Humanitas Research Hospital, Rozzano, Milan Italy; 2 Department of Biomedical Sciences, Humanitas University, Pieve Emanuele, Milan, Italy; 3 MOX Laboratory, Department of Mathematics, Politecnico di Milano, Piazza Leonardo da Vinci 32, 20133 Milan, Italy; 4 Department of Nuclear Medicine, IRCCS Humanitas Research Hospital, Milan, Italy; 5 CHDS - Center for Health Data Science, Human Technopole, Milan, Italy; 6 Hepatobiliary Unit, Department of Minimally Invasive General & Oncologic Surgery, Humanitas Gavazzeni University Hospital, Via M. Gavazzeni 21, 24125 Bergamo, Italy

**Keywords:** Texture analysis, Entropy, Colorectal liver metastases, Radiomics, CT scan, Quantitative imaging

## Abstract

Advanced imaging and analysis improve prediction of pathology data and outcomes in several tumors, with entropy-based measures being among the most promising biomarkers. However, entropy is often perceived as statistical data lacking clinical significance. We aimed to generate a voxel-by-voxel visual map of local tumor entropy, thus allowing to (1) make entropy explainable and accessible to clinicians; (2) disclose and quantitively characterize any intra-tumoral entropy heterogeneity; (3) evaluate associations between entropy and pathology data. We analyzed the portal phase of preoperative CT of 20 patients undergoing liver surgery for colorectal metastases. A three-dimensional core kernel (5 × 5 × 5 voxels) was created and used to compute the local entropy value for each voxel of the tumor. The map was encoded with a color palette. We performed two analyses: (a) qualitative assessment of tumors’ detectability and pattern of entropy distribution; (b) quantitative analysis of the entropy values distribution. The latter data were compared with standard Hounsfield data as predictors of post-chemotherapy tumor regression grade (TRG). Entropy maps were successfully built for all tumors. Metastases were qualitatively hyper-entropic compared to surrounding parenchyma. In four cases hyper-entropic areas exceeded the tumor margin visible at CT. We identified four “entropic” patterns: homogeneous, inhomogeneous, peripheral rim, and mixed. At quantitative analysis, entropy-derived data (percentiles/mean/median/root mean square) predicted TRG (*p* < 0.05) better than Hounsfield-derived ones (*p* = n.s.). We present a standardized imaging technique to visualize tumor heterogeneity built on a voxel-by-voxel entropy assessment. The association of local entropy with pathology data supports its role as a biomarker.

**Graphical Abstract:**

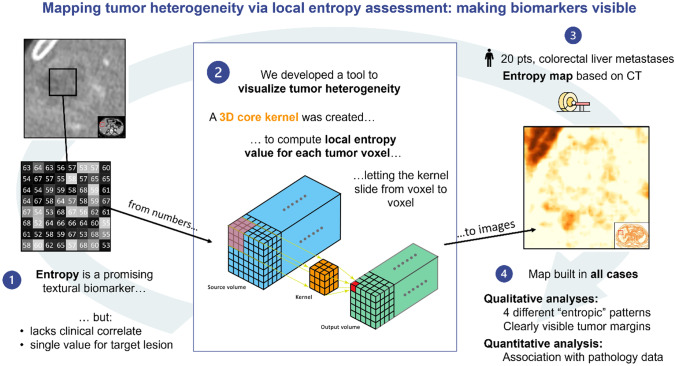

**Supplementary Information:**

The online version contains supplementary material available at 10.1007/s10278-023-00799-9.

## Background

In recent years, technological progresses have favored advanced analyses applied to medical imaging [1, 2]. There is indeed thriving literature on the contribution of texture analysis, i.e., radiomics, to the prediction of pathology data and outcome in several tumor types [2–4]. Entropy has emerged as one of the most relevant radiomic features. It measures the information content: regions requiring more information to be described present higher values of entropy. In many series, this index has been associated with tumor aggressiveness [3, 5, 6]. Nevertheless, the translation of entropy into clinical practice is still underway, first and foremost because of its “intangible” nature and the lack of an exact pathology correlate. Furthermore, the conventional radiomic frameworks for feature extraction provide a single value of entropy for the whole lesion, missing any intra-tumor heterogeneity.

To overcome those limitations, we explored a novel visualization tool, capable of providing a voxel-by-voxel map of the local entropy across a tumor area, to visualize intra-tumoral heterogeneity. This study aimed to (1) make entropy accessible by visual inspection; (2) identify and quantitively characterize any intra-tumoral entropy heterogeneity; and (3) provide a preliminary evaluation of the association between local entropy and pathology data.

## Methods

### Study Population

We considered all consecutive patients that underwent surgical resection for colorectal liver metastases (CLM) at our institution between June 2017 and December 2020. The following inclusion criteria were used: age ≥ 18 years; CT scan performed ≤ 30 days before surgery at the authors’ institution; adequate portal phase of the CT scan; CLM with diameter ≥ 10 mm; preoperative chemotherapy. The CT acquisition was performed according to a standardized protocol, as previously described [7]. Lesions < 10 mm were excluded because they could not guarantee enough voxels for the analysis. In patients with multiple CLMs, the largest lesion was analyzed. This retrospective study was approved by the local ethics committee (protocol #83/20) and the need for specific informed consent was waived.

### Treatment Response Assessment

Treatment response to chemotherapy was radiologically and pathologically assessed according to the RECIST [8] and the Rubbia-Brandt criteria [9], respectively. Considering the pathological response (i.e., the tumor regression grade, TRG), the patients were classified as responders (TRG 1–3) and non-responders (TRG 4–5).

### Creation of the Entropy Maps

**Fig. 1:**
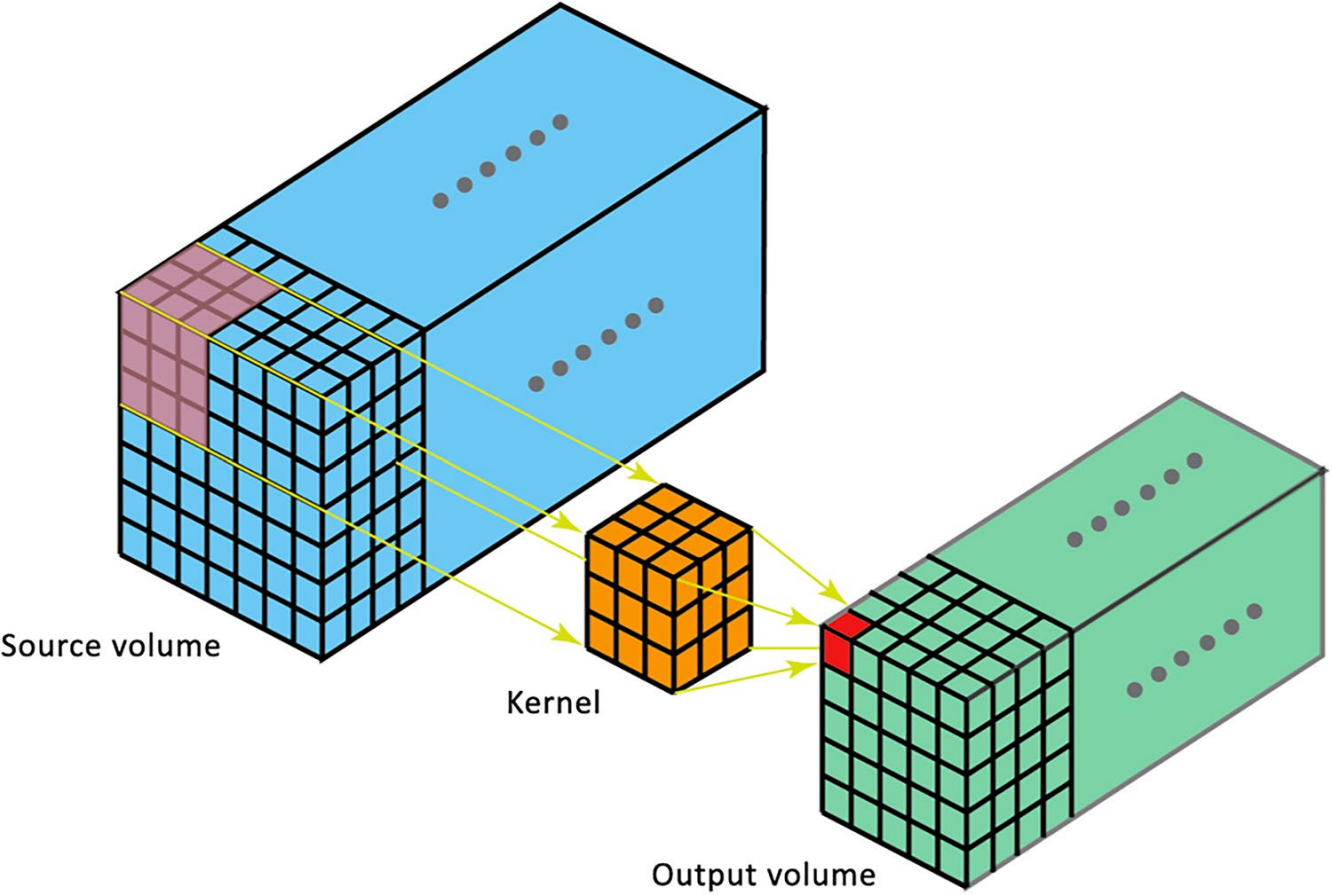
Building of the entropy maps. Slices including the liver were selected on the portal phase of the pre-operative CT scan (blue volume). A three-dimensional kernel (5 × 5 × 5 voxels) was considered for the computation of the local entropy (orange cube). The entropy value was retained and saved in the map as the focal entropy of the central voxel of the kernel (red cube). This procedure was repeated for every voxel of the volume, letting the kernel slide with a one-voxel stride, and obtaining an output volume (green volume)

For each patient, a tumor entropy map was built according to the following steps:

1. All slices of the portal phase of preoperative CT were considered. (Fig. 1, blue volume).
2. A three-dimensional kernel with a dimension of 5 × 5 × 5 voxels (Fig. 1, orange cube) was used for the computation of local entropy. A sensitivity analysis of the impact of different kernel dimension $(k)$ on the local entropy map computation and performance was performed (Supplementary Fig. 1). The 5 × 5 × 5 one was chosen as the best trade-off between spatial resolution and noise smoothing.

Within each kernel, the computation of entropy was performed according to the following formula:

$$Entropy = -\sum\limits_{\left\{i=1\right\}}^{n}P\left(i\right){\mathrm{log}}_{e}(P(i))$$

with “P” being the probability of occurrence of each of n voxel intensity “i” in the volume.

**Fig. 2:**
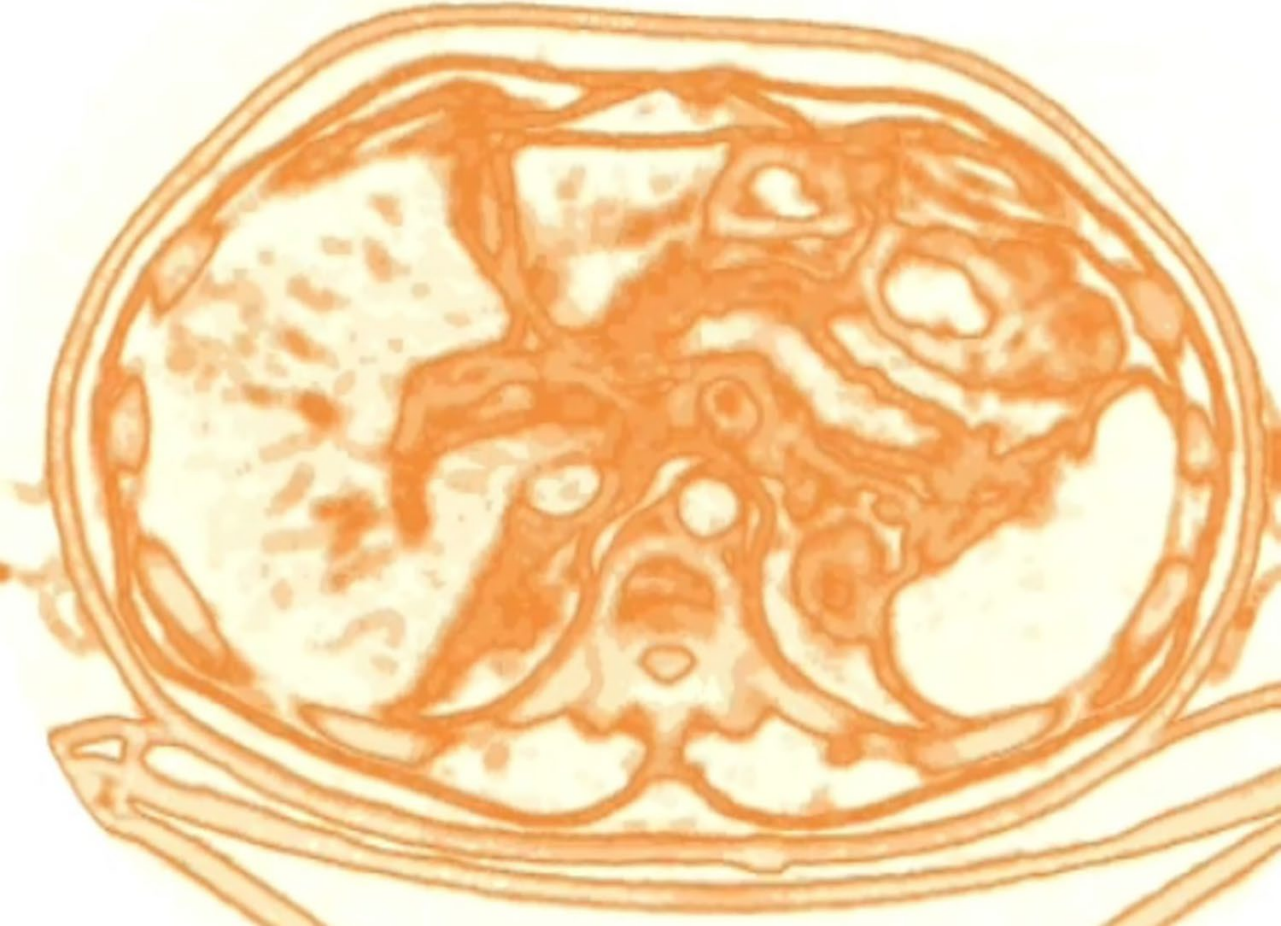
The entropy map: example of a slice. The three-dimensional entropy map obtained after the computation process was then sliced to provide images similar and comparable to CT images

3. The so-computed value was retained and saved in the map as the local entropy of the central voxel of the kernel (Fig. 1, red cube). This procedure was repeated for every voxel of the volume, letting the kernel slide with a one-voxel stride. The resulting volume was defined as the tumor entropy map (Fig. 1, green volume).
4. The map was obtained encoding the range of the entropy values with a dedicated color palette. To optimize the visualization of the tumor entropy, slices were smoothed with a Gaussian filter with $\sigma =1$ and normalized over the mean entropy value of the patient’s liver parenchyma.
5. The resulting three-dimensional entropy map was sliced to provide images similar and comparable to CT images. This allowed a visual assessment of the distribution of entropy, with the identification of “hyper-entropic,” “iso-entropic,” and “hypo-entropic” areas, in comparison with the liver parenchyma remote from the tumor (Fig. 2).

### Comparison of CT Images and Entropy Maps

Two expert readers with long-standing experience in advanced image analysis (GC and FF), under the supervision of an MD expert in radiology (AC), executed a slice-by-slice manual segmentation of the CLM (Tumor-VOI) on the same CT slices used for the creation of the entropy map. The Tumor-VOI was then superimposed to the entropy map to match the manually segmented lesion with the one resulting from the entropy map.

### Data Analysis

We performed two separate analyses. First, we qualitatively analyzed the entropy maps evaluating the characteristics of the tumor and peritumoral liver parenchyma (hyper/hypo/iso-entropic), the detectability of the tumor and its margin, the pattern of entropy distribution into the tumor, and the visual match between the entropy-based tumor area and the manually segmented Tumor-VOI.

Second, we performed a quantitative analysis of the distribution of the entropy values by extracting their histogram from each map. We considered the portion of the entropy map delimited by the superimposed Tumor-VOI. The following descriptive statistics were computed: mean, median, standard deviation, variance, root mean square (RMS), percentiles (5th, 25th, 75th, 95th), number of zero crossings, and number of mean crossings. In addition, we used the LifeX software [10, 11] to compute the global entropy of the tumor (Tumor-VOI) and the entropy of the non-tumoral liver parenchyma. For the latter, we performed a virtual liver biopsy (Liver-VOI), as previously detailed [12]. Upon *z*-score standardization, univariate non-parametric statistical tests were used to compare variables’ distribution in responders (TRG 1–3) and non-responders (TRG 4–5). According to the literature data demonstrating an association between the Hounsfield units and tumor response to chemotherapy [13, 14], the same processing workflow was carried out on CT images: the histogram of the Hounsfield units was extracted from each Tumor-VOI, and the descriptive statistics were compared between responders and non-responders. A *P*-value < 0.05 was considered significant for all tests.

## Results

Our cohort included 20 patients, twelve males and eight females, with a median age of 63 years (range, 41–83). All had preoperative chemotherapy. The median CLM size before surgery was 17 mm (range, 10–57 mm). According to the RECIST criteria, 13 patients had a partial response and 7 a stable disease. According to the pathology data, 8 patients were responders (TRG 1–3) and 12 non-responders (TRG 4–5). Five of the 13 (38%) patients having a partial response at imaging did not have a tumor response at pathology (TRG 4–5). The entropy map was obtained in all cases. Table 1 summarizes the patients’ data.

**Table 1:** Patient’s characteristics and entropy patterns of the metastases at the qualitative analysis

**Demographics and tumor characteristics**	*N* (%) – Median (range)
Age, years	63 (41–83)
Sex (M/F)	12 (60): 8 (40)
Primary tumor	
Colon/rectum	15 (75) / 5 (25)
T3/4	16 (80)
N+	15 (75)
Liver metastases	
Size, mm	17 (10–57)
Synchronous disease	15 (75)
Strategy in synchronous metastases	
Colon-first approach	10/15 (67)
Liver-first approach	3/15 (20)
Simultaneous hepatic/colo-rectal resection	2/15 (13)
Preoperative chemotherapy	20 (100)
Oxaliplatin	13 (65)
Irinotecan	6 (30)
Oxaliplatin + Irinotecan	1 (5)
+Anti-VEGF	5 (25)
+Anti-EGFR	4 (20)
Cycles of chemotherapy	7 (4–32)
Radiological response	
Stable disease	7 (35)
Partial response	13 (65)
Pathological response	
TRG 1–2	7 (35)
TRG 3	1 (5)
TRG 4–5	12 (60)
**Qualitative analysis**	
**Entropy pattern**	
Homogeneous pattern	3 (15)
Inhomogeneous	5 (25)
Peripheral rim	9 (45)
* Complete*	3 (15)
* Incomplete*	6 (30)
Mixed pattern	3 (15)

### Qualitative Analysis

**Fig. 3:**
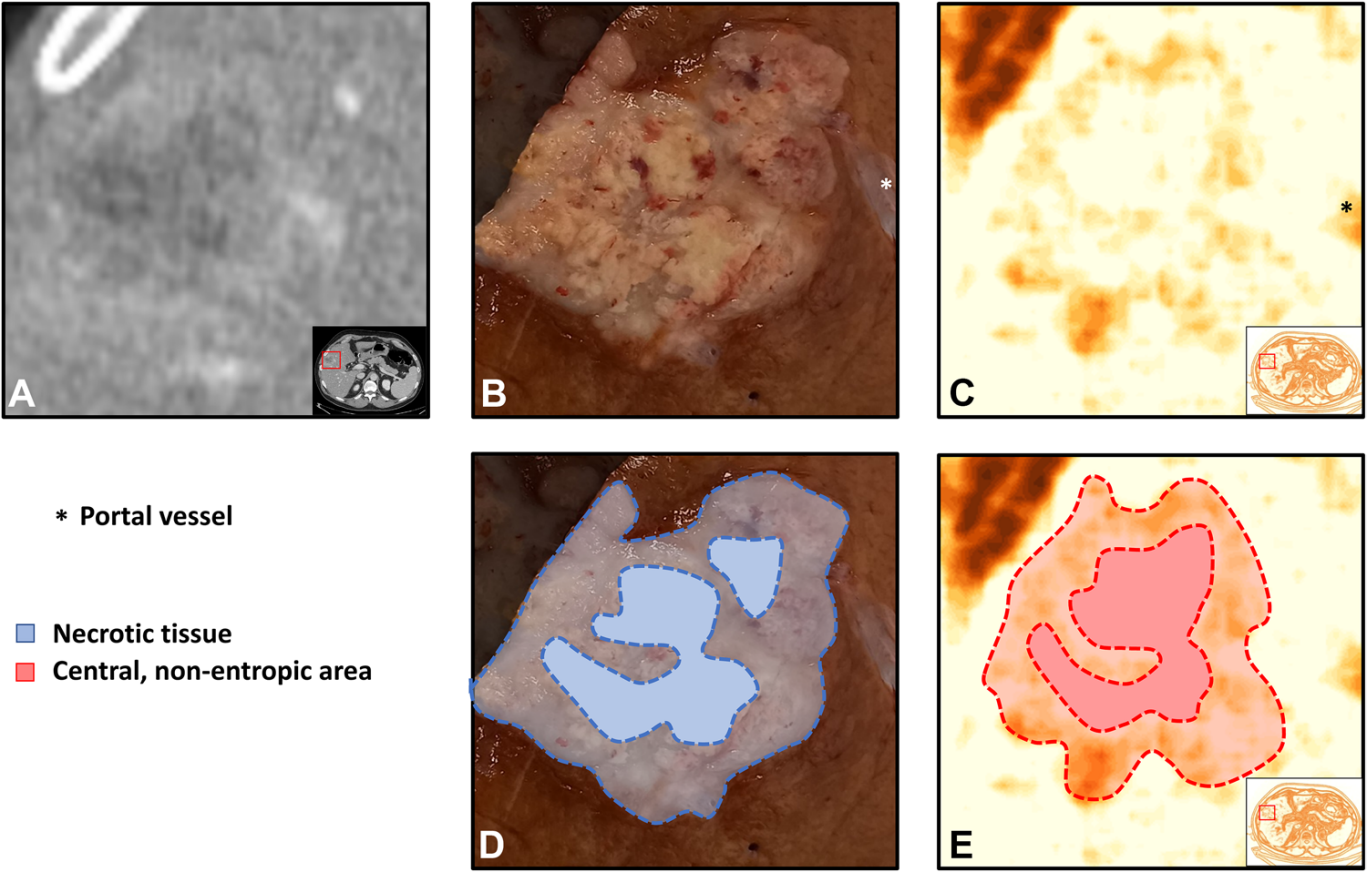
Comparison of the resection specimen (**B**, **D**), CT scan (**A**), and entropy maps (**C**, **E**). A patient with a colorectal liver metastasis located in segment 5. The entropy map provides accurate identification of the tumor edges and necrotic tissue

**Fig. 4:**
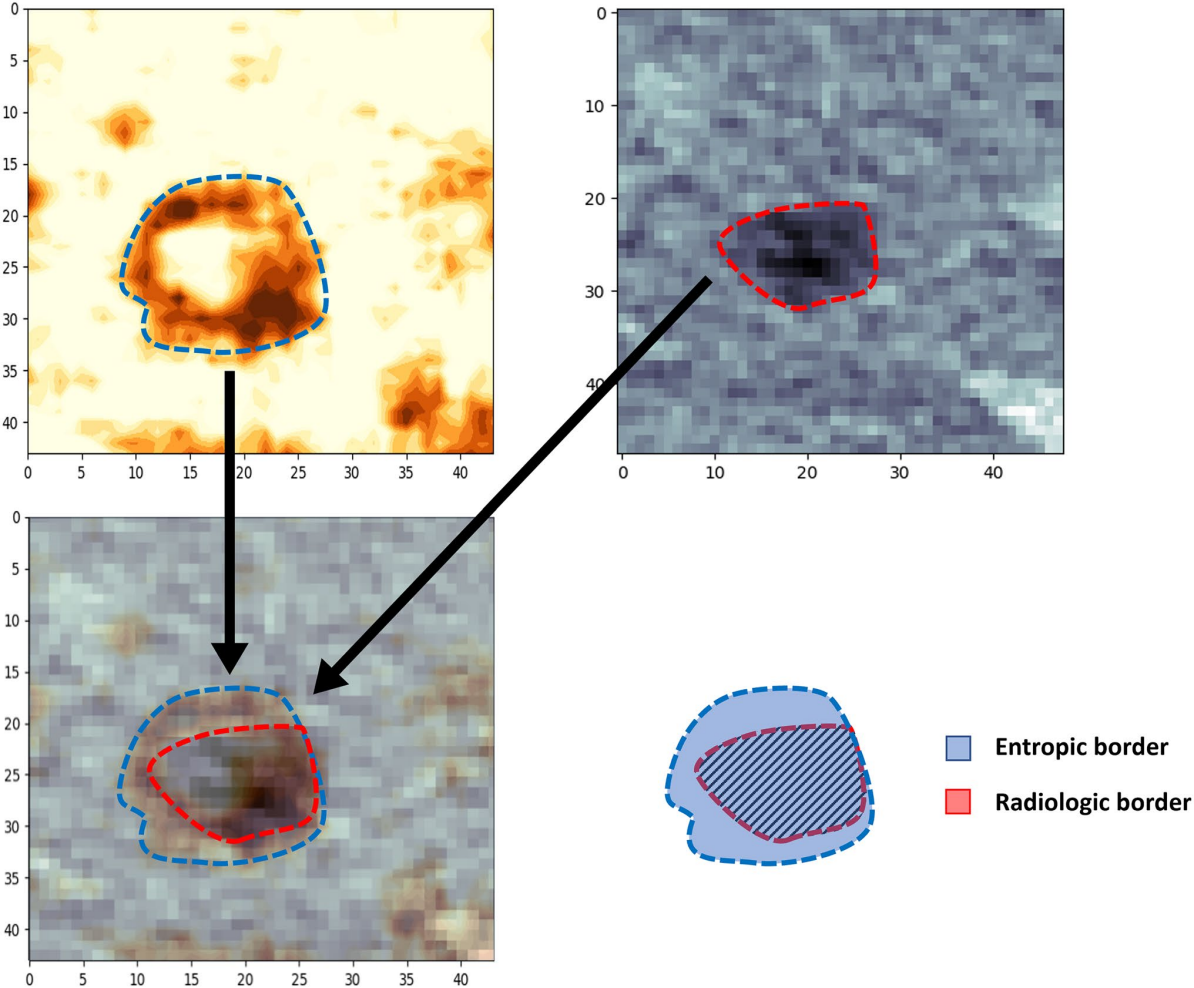
Discrepancies of the tumor boundaries between CT scan and entropy map. In a colorectal liver metastasis located in segment 8, the entropy map showed “hyper-entropic” areas extending beyond the border of the lesion detected on the CT scan

The entropy maps identified all CLMs (CLMs presented at least some *foci* of “hyper-entropic” tissue) and provided accurate identification of the tumor edges (Figs. 3 and 4). The non-tumoral liver parenchyma had homogeneous entropy. Intrahepatic small vessels were uniformly “hyper-entropic,” while large ones (e.g., hepatic veins at their caval confluence) were “hypo-entropic” with a “hyper-entropic” rim.

**Fig. 5:**
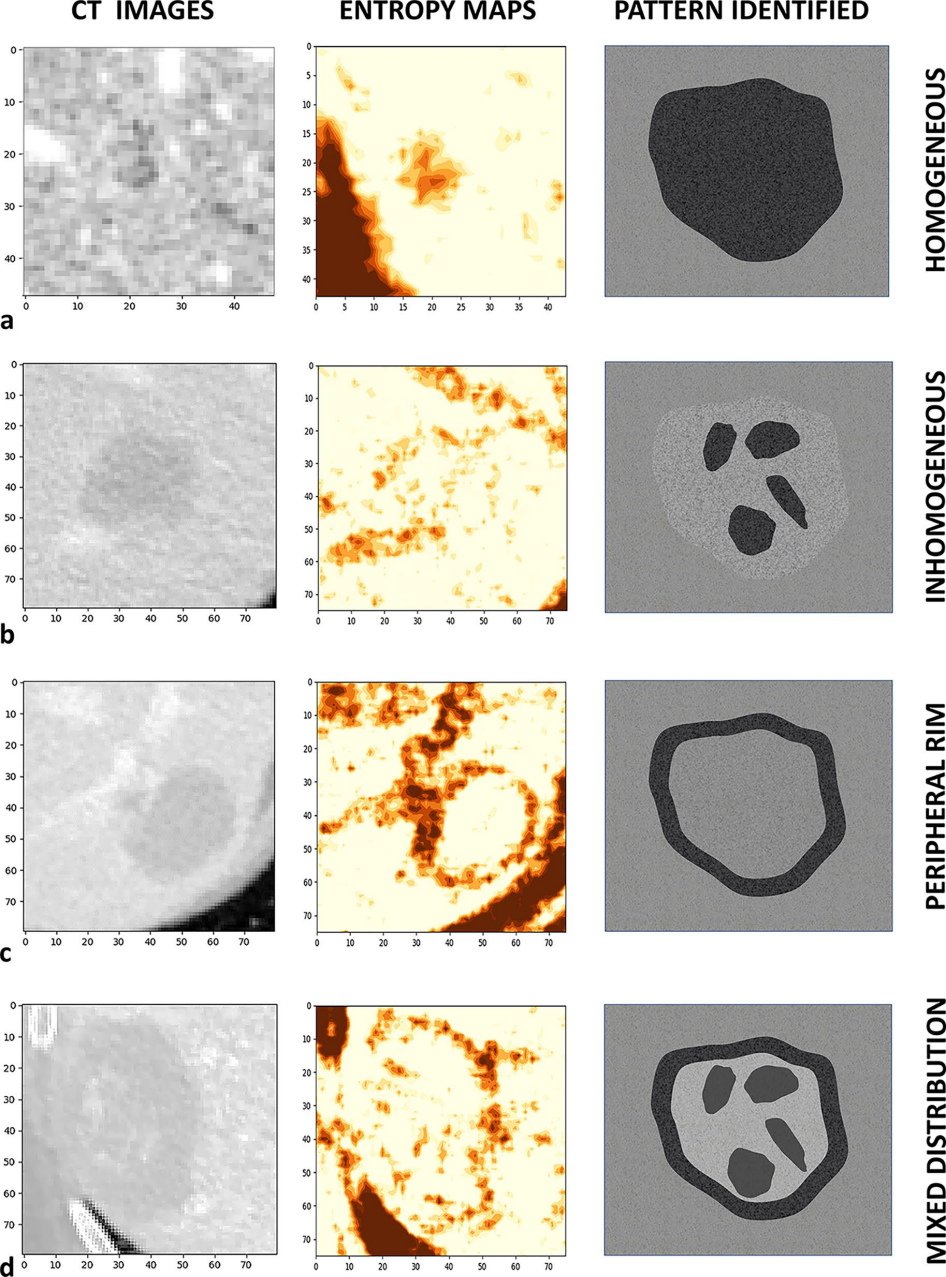
Patterns of entropy distribution into the tumors. After qualitative analysis, four different patterns of distribution of entropy were identified: **a** homogeneous; **b** inhomogeneous; **c** peripheral rim; **d** mixed distribution. CT scan (first column), entropy map (second column), and schematic representation of the patterns are reported

The entropy of CLMs differed among patients. We identified four “entropic” patterns (Fig. 5):

- Homogeneous pattern: the lesion appears as a uniform “hyper-entropic” tissue with a homogeneous distribution of entropy in ≥ 75% of its surface.
- Peripheral rim pattern: a peripheral “hyper-entropic” rim surrounds a homogeneous central “iso/hypo-entropic” area for ≥ 75% of its circumference. The rim can be complete or incomplete. The central area is homogeneous, i.e. ≥ 75% of its surface has a uniform entropy.
- Inhomogeneous pattern: the lesion has a scattered distribution of “hyper-” and “iso/hypo-entropic” components (hyper-entropic areas < 75% of the surface) and does not have a peripheral rim.
- Mixed pattern: the lesion has a peripheral “hyper-entropic” rim in combination with an inhomogeneous core, as defined above.

Patterns are detailed in Table 1. The most common pattern was the peripheral rim (9 out of 20 cases), while the homogeneous one was detected only among the smallest metastases (size < 15 mm).

Superimposing the manually segmented Tumor-VOI to the entropy map, we observed some “hyper-entropic” areas extended beyond the Tumor-VOI contour in four cases (Fig. 4).

### Quantitative Analysis

The global entropy of the Tumor-VOI computed with LifeX was 0.91 ± 0.08, higher than the entropy of the non-tumoral liver parenchyma (Liver-VOI, 0.75 ± 0.09, *p* < 0.001). Responders (TRG 1–3) had higher global entropy values than non-responders (0.96 ± 0.06 vs 0.88 ± 0.09; *p* = 0.020).

**Fig. 6:**
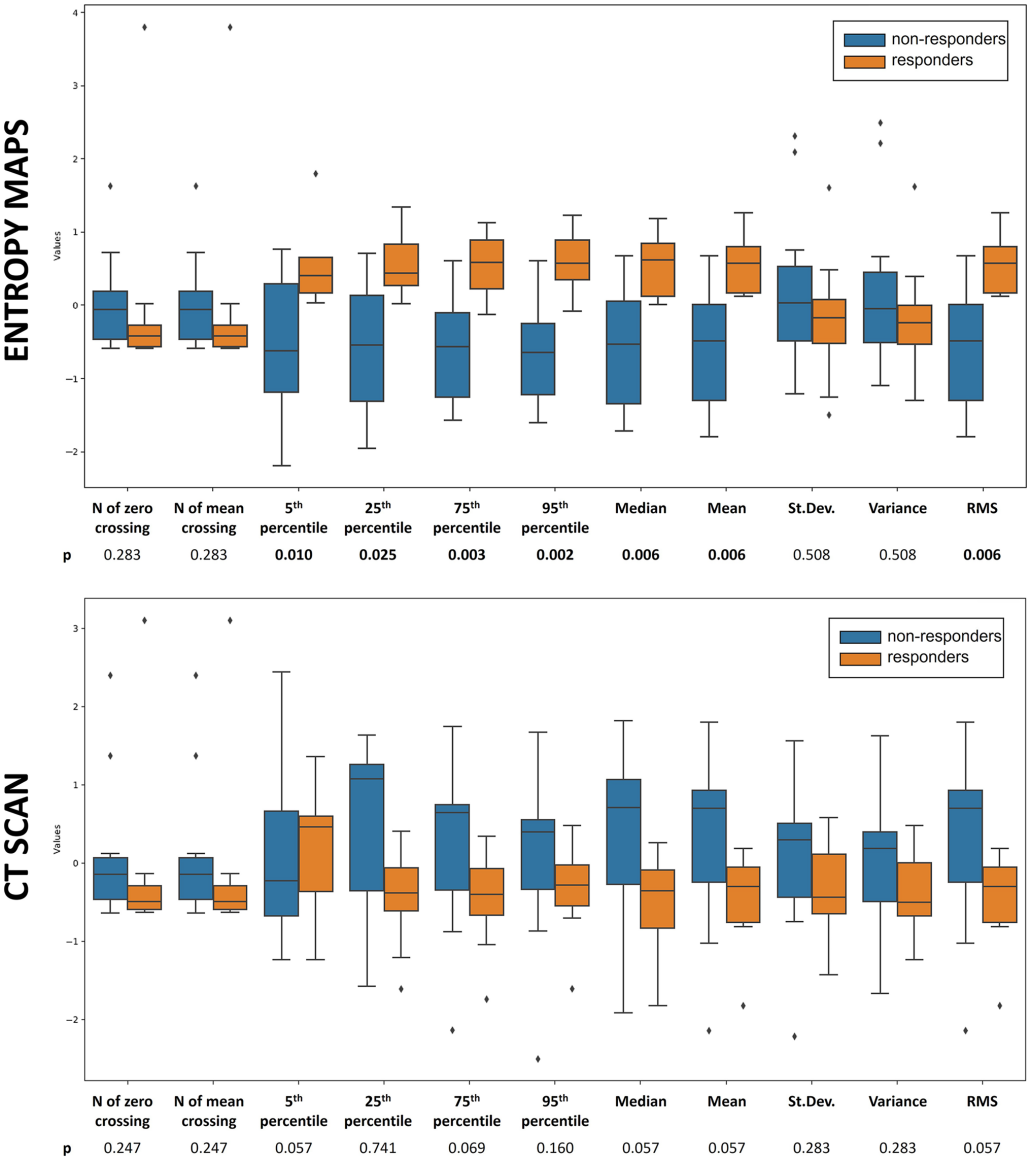
Results of quantitative analysis. Analysis of the distributional variables extracted from the histogram of CT scan (Hounsfield-derived, lower box) and entropy maps (upper box). Patients with and without pathological tumor response to chemotherapy were compared (responders, TRG 1–3, orange bars; non-responders, TRG 4–5, blue bars). Seven variables extracted from the histograms of entropy maps were significantly associated with the response to chemotherapy. No Hounsfield-derived variable was associated with the response to chemotherapy. *RMS, root mean square*

Of the 11 variables extracted from the histograms of the entropy maps, seven were associated with TRG (percentiles, 5th, *p* = 0.025; 25th, *p* = 0.010; 75th, *p* = 0.003; 95th, *p* = 0.002; median, p = 0.006; mean, p = 0.006; RMS, p = 0.006), with stronger correlation than global Tumor-VOI entropy. In detail, magnitude variables showed higher importance than variability indices, with higher values characterizing responders over non-responders. Considering the histograms of the Hounsfield units extracted from each Tumor-VOI, no Hounsfield-derived variable was associated with the TRG. Data are summarized in Fig. 6 and Table 2.

**Table 2:** Analysis of the occurrence-based variables extracted from the histogram of the entropy maps. Patients with and without pathological tumor response to chemotherapy (TRG1-3 vs. TRG 4–5) were compared

**Quantitative analysis**	**Whole series**	**Responders** **TRG 1–3**	**Non-responders** **TRG 4–5**	***P***
**Standard entropy measurement (whole tumor)**				
Global entropy	0.91	0.96	0.88	0.020
**Entropy map measurement**				
Mean (Std. Dev.)	3.65 (0.12)	3.71 (0.11)	3.59 (0.12)	0.006
Median	3.66	3.71	3.59	0.006
Variance	0.014	0.013	0.015	0.508
5th percentile	3.45	3.51	3.39	0.010
25th percentile	3.58	3.63	3.51	0.025
75th percentile	3.73	3.79	3.67	0.003
95th percentile	3.84	3.89	3.79	0.002
Root mean square—RMS	3.65	3.71	3.59	0.006
Number of (#) zero crossing	5674.81	6734.50	5863.27	0.283
Numbero of (#) mean crossing	5674.81	6734.50	5863.27	0.283

## Discussion

The present manuscript depicts a novel approach to advanced image analysis, aiming at the visualization of tumor entropy. The procedure was successfully applied to all patients. We were able to correctly identify the tumor and its margin within the entropy maps. In some cases, we even identified a discrepancy between the tumor-contour detected by the entropy maps and the one visible at CT. Entropy within the lesion was irregularly distributed and had different patterns. Finally, the quantitative analysis of the map showed considerable discriminant power of entropy distribution—better than standard texture analysis or conventional parameters—in predicting pathology data (i.e., TRG).

The textural analysis represents a major achievement of modern medical imaging. In several tumors, it allows the identification of clinically relevant biomarkers, based on invisible-to-eye pixel and voxel patterns, which improve the prediction of pathology and outcome data [1, 2]. Entropy is one of the most investigated textural features and, in CLMs, has been associated with radiological response to chemotherapy as well as prognosis [3, 15–18]. Nevertheless, radiomic features did not impact clinical practice yet because they are still felt as statistical and mathematical data lacking an immediate clinical significance. While the heterogeneity in the greyscale of Hounsfield units can be easily appreciated, the entropy remains an abstract concept, even if it can catch some microscopic characteristics missed by standard imaging modalities [7]. We tried to overcome this limitation by building a colored map of entropy, disclosing differences between the tumor and liver parenchyma, and unveiling its intra-tumoral distribution.

In our pilot series, both the qualitative and quantitative information showed a potential connection with the clinical and pathological domains. The visual inspection of the entropy maps allowed to identify the tumor in all patients as a “hyper-entropic” volume, which, in some cases, exceeded the tumor margins identified on the CT. The latter finding could correspond to well-known pathological data, i.e., the early regrowth of the metastases at their periphery after chemotherapy and the peritumoral micrometastases, which both dictate the need for a wide surgical margin [19, 20]. Furthermore, at qualitative analysis, we were able to characterize intra-tumoral heterogeneity and catch focal levels of disorder: different entropic patterns emerged, even if the sample of patients was small. These patterns deserve further investigation for their potential correspondence with a pathology/molecular profile. For instance, the peripheral rim might reflect the liver-tumor interface, which is the niche of major biomarkers (e.g., the tumor growth pattern and peritumoral immune infiltrate) and the battlefield for tumor progression [21–23]. Different patterns could correspond to different tumor behavior requiring different surgical and oncological strategies. Intrahepatic vessels showed increased entropy as the tumor did but they can be easily identified by comparing the entropy map with the portal venous CT. In the future, the task of removing the vessel-related entropy component could be even automated.

Considering the quantitative analyses, the voxel-by-voxel evaluation of entropy provided relevant information. We performed a preliminary evaluation of the potential clinical relevance of the current study by assessing the association between the entropy maps and TRG. In the literature, RECIST criteria have limited capability to predict the pathological response to chemotherapy [20, 24], while entropy [3, 15–18] and tumor density (Hounsfield units) [13, 14] achieve better performances. The present study not only confirmed the poor reliability of the RECIST criteria but also demonstrated that a detailed analysis of the intra-tumoral entropy maximizes the prediction of response, improving the one achieved by a single global entropic value and by Hounsfield units. A more accurate depiction of entropy has the potential to grant a deeper comprehension of tumor biology.

We herein introduce a user-friendly approach to radiomic analyses with their visual representation. It can be theoretically applied to all textural features, to different imaging modalities, and to different tumors, not only the hepatic ones. The entropy maps could be superimposed to the CT and read in fusion mode, as currently done for PET-CT. Several clinical implications can be anticipated. We moved the task of radiomics interpretation within a “comfort zone” for the clinicians, making the spatial heterogeneity of entropy discernible. This opens the way to an easy association of textural features with the pathology data and could help modern oncology to pursue a precision medicine approach. In fact, the non-invasive assessment of tumor heterogeneity is crucial to optimize and personalize the treatment strategy [25, 26] but is still an unmet need. The quantitative analysis of the map could provide new radiomic-based biomarkers. The analysis of liver entropy could even be used to spot lesions in their earliest stages when they have not caused structural changes yet. Despite the promising results of our study, some limitations have to be acknowledged: reproducibility is still under testing; clinical implications remain to be proved; further investigations with more complex models and a larger cohort of subjects are needed. Moreover, the integration of further parameters, such as tumoral perfusion [27], and a dynamic assessment of entropy, e.g., before and after systemic therapy, could improve the capability of the method to stratify the lesions even further.

## Conclusion

In conclusion, this approach could represent the new frontier of non-invasive evaluation of tumor biology and be a crucial step toward the clinical application and interpretation of radiomics.

## Supplementary Information

Below is the link to the electronic supplementary material.

Supplementary Figure 1. Comparison in terms of mean and standard deviation of the performance indices for different kernel dimension (k) values in Local Entropy Map (LEM) computation. Specifically, **a** time demand, **b** resources demand, **c** tumor grade regression performance and **d** response to therapy prediction performance are considered. (DOCX 96 KB)

## Data Availability

Data are available and can be obtained from the corresponding author upon reasonable request.

## Abbreviations

CLM: Colorectal liver metastases
TRG: Tumor regression grade
RMS: Root mean square
VOI: Volume of interest
