# Supplementary material for: Mapping Tumor Heterogeneity via Local Entropy Assessment: Making Biomarkers Visible
Source: J Digit Imaging. 2023 Feb 27;36(3):1038–48. doi: 10.1007/s10278-023-00799-9 (PMC10287605; doi:10.1007/s10278-023-00799-9)
Supplement: Supplementary file 1 — Supplementary Figure 1. Comparison in terms of mean and standard deviation of the performance indices for different kernel dimension (k) values in Local Entropy Map (LEM) computation. Specifically, a time demand, b resources demand, c tumor grade regression performance and d response to therapy prediction performance are considered. (DOCX 96 KB) [file 10278_2023_799_MOESM1_ESM.docx]

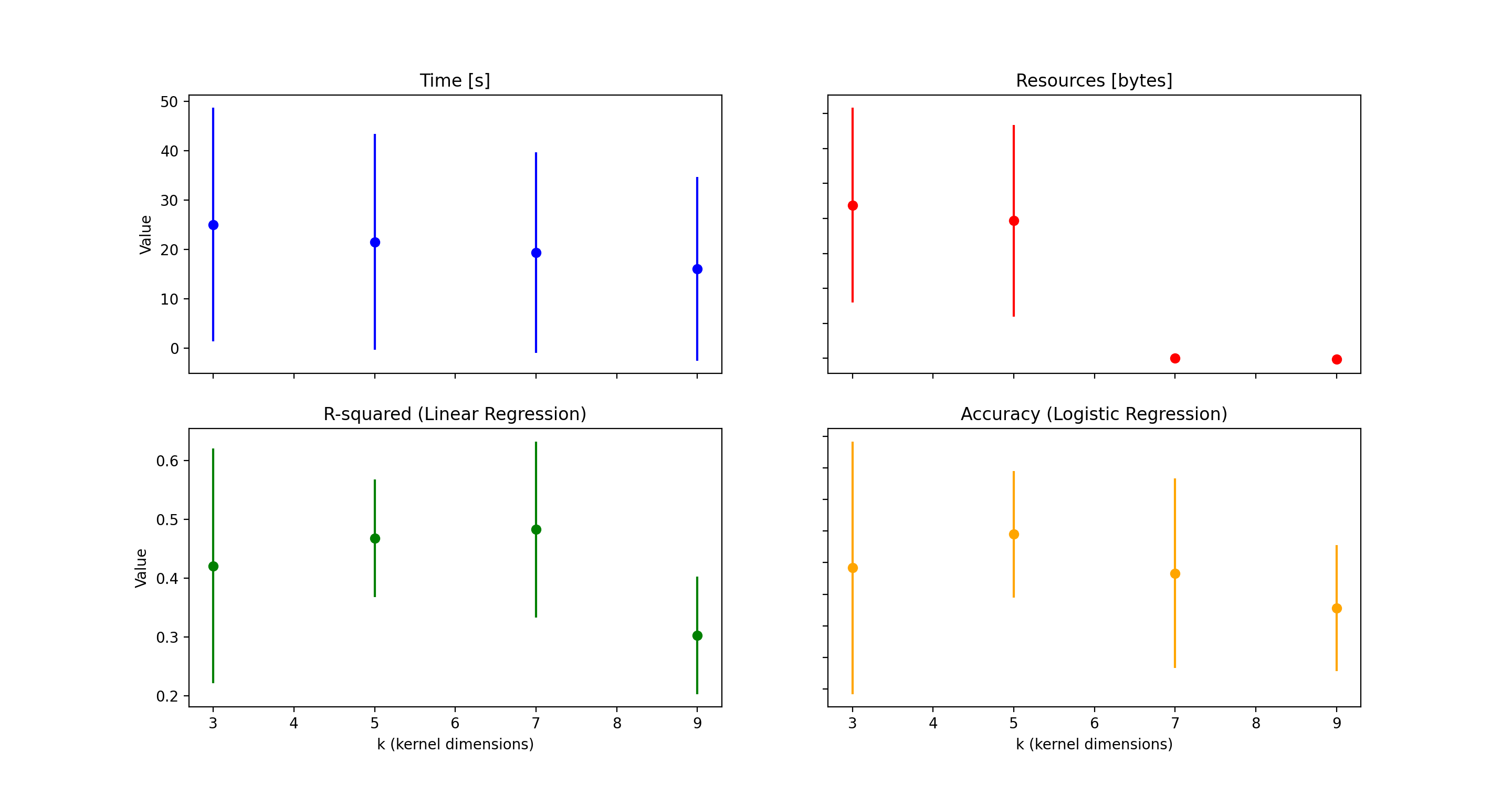


**Supplementary Figure 1**. Comparison in terms of mean and standard deviation of the performance indices for different kernel dimension (k) values in Local Entropy Map (LEM) computation. Specifically, a) time demand, **b**) resources demand, **c**) tumor grade regression performance and **d**) response to therapy prediction performance are considered.
